# Help Seeking and Access to Primary Care for People from “Hard-to-Reach” Groups with Common Mental Health Problems

**DOI:** 10.1155/2011/490634

**Published:** 2011-07-06

**Authors:** K. Bristow, S. Edwards, E. Funnel, L. Fisher, L. Gask, C. Dowrick, C. Chew Graham

**Affiliations:** ^1^Mental Health and Wellbeing, Institute of Psychology, Health and Society, University of Liverpool, Waterhouse Building, Liverpool L69 3GL, UK; ^2^School of Community-Based Medicine, Primary Care Research Group and National School of Primary Care Research, University of Manchester, Williamson Building, Manchester M13 9PL, UK

## Abstract

*Background*. In the UK, most people with mental health problems are managed in primary care. However, many individuals in need of help are not able to access care, either because it is not available, or because the individual's interaction with care-givers deters or diverts help-seeking. *Aims*. To understand the experience of seeking care for distress from the perspective of potential patients from “hard-to-reach” groups. *Methods*. A qualitative study using semi-structured interviews, analysed using a thematic framework. *Results*. Access to primary care is problematic in four main areas: how distress is conceptualised by individuals, the decision to seek help, barriers to help-seeking, and navigating and negotiating services. *Conclusion*. There are complex reasons why people from “hard-to-reach” groups may not conceptualise their distress as a biomedical problem. In addition, there are particular barriers to accessing primary care when distress is recognised by the person and help-seeking is attempted. We suggest how primary care could be more accessible to people from “hard-to-reach” groups including the need to offer a flexible, non-biomedical response to distress.

## 1. Introduction

According to the World Health Organization, half of all people with ill health in Western Europe have mental illness, with the majority coming into the diagnostic categories of anxiety and depression [1]. Such problems impose substantial emotional, social and economic burdens on those who experience them, their families and carers, and society as a whole [2].

The National Service Framework for mental health in England [3] dictated that the majority of patients with common mental health problems should be managed in primary care. Currently 90% of people with mental health problems in the UK are managed by GPs. Primary care provides the first point of contact for many people and also act as a gatekeeper to other statutory services. GPs can refer patients to primary care mental health teams for short-term psychosocial interventions, but in reality have limited access to secondary care services [4]. 

The UK's National Health Service (NHS) states it has an explicit access-driven health policy framework [5–8], yet there is evidence that individuals with high levels of mental distress may be unable to access care, either because it is not available or because the individual's interaction with care-givers deters or diverts help-seeking [9]. This is the premise of the AMP study (“AMP—Improving Access to Primary Mental Health Care” in Liverpool and Manchester) [10], a National Institute for Health Research funded project (RP-PG-0606-1071). The aim of AMP is to increase equity of access to high quality mental health services in primary care. Groups with inadequate access to primary care include people, from black and minority ethnic (BME) communities, asylum seekers, homeless people and adolescents with eating disorders [11–13]. Groups who receive inadequate help when they do access primary care include elders, people with advanced cancers, those at risk of long-term sickness absence, and people with medically unexplained symptoms (MUS) [14–16].

As outlined by Kovandžić et al. [17], research on access has been driven by two different disciplinary perspectives. Firstly, health services research has traditionally examined access to health care from the point of entrance to the formal system of care. The focus here is on supply-side factors, the availability of treatments, and on structural and organisational change to remove barriers, rather than on demand issues governing the journey of the patient in need. Key concepts such as demand, availability, utilization, and patterns of use are developed in a functionalist view of the relation between service provision and use [18]. In addition, the literature has tended to focus on whether mental health issues are recognised by primary care practitioners.

Secondly, behavioural and social science traditions focus mainly on an “out-of-service” perspective, for example, on processes that happen before the point of entrance into formal systems of care, formulating the scope of research into an umbrella term of “help-seeking”. Broadhurst [19] identifies a series of three-stage models of help-seeking, summarizing their variations in (a) problem definition, (b) deciding to seek help, and (c) actively seeking help. Access is conceptualised as an interaction between supply and demand of services mediated and codified by professionally defined needs for services. Dixon-Woods et al. [18] describe a model of access which attempts to explain where barriers might be for people seeking care. *Candidacy* captures how people's eligibility for healthcare is determined by the interaction between patients and health services. Following identification of candidacy, individuals attempt *navigation* and *negotiation *to gain a point of entry to health services. Rogers et al.'s. concept of *recursivity* refers to the interdependency between a user's experiences of health services and her/his future actions in regards to health and help seeking [20]. A further term, *concordance, *was used by Stevenson and Scambler [21] to convey the need for the help seeker and the practitioner to find common ground in terms of what the problem and its subsequent solutions might be. Concepts such as candidacy, navigation, recursivity, and concordance imply that user perspectives and their interaction with clinicians in primary care are vital to understand the type of care given and received and its influence on future help-seeking. 

Most of the literature around detection of mental health problems in primary care relates to depression and for over 40 years, GPs have been told that they fail to diagnose depression [22, 23]. Some studies, however, indicate that [23, 24] clinically significant depression (moderate to severe depressive illness) is detected by GPs at later consultations by virtue of the longitudinal patient-doctor relationship and it is milder forms, which may recover spontaneously, that go undetected and untreated. Some authors draw attention to the dangers of the erroneous diagnosis of depression in patients with a slight psychological malaise and little functional repercussion leading to the risk of unnecessary and potentially dangerous medicalisation [25, 26]. Detection of depression may be poor if primary care clinicians lack the necessary consultation skills or confidence to make the diagnosis correctly. Initiatives to improve GPs' skills in the detection and management of depression have been evaluated [27] but such interventions alone have not led to improvements in patient outcome. 

There is only a limited literature considering the patient perspective and this focuses on the limited value of Western approaches to mild and moderate depression in patients of different ethnic groups. This literature indicates that people may have difficulty in presenting their distress and discussing their concerns with their doctor, especially when they are uncertain that depression is a legitimate reason for seeing the doctor [28].

Some people consider that the GP is not the most appropriate person to talk to or believe that symptoms of distress should not be discussed at all in the primary care consultation. Others feel that they do not deserve to take up the doctor's time or that it is not possible for doctors to listen to them and understand how they feel [29]. Whilst previous literature attempts to explain the difficulties people from hard-to-reach groups have in dealing with mental health problems, they focus on understandings of mental illness, rather than *access* per se. This paper aims to explore help-seeking and access to care from the perspectives of potential patients from “hard-to-reach” groups.

## 2. Methods

The data for this paper are derived from a qualitative study within the AMP programme. Ethical approval was granted by Wrightington, Wigan and Leigh Research Ethics Committee (Reference: 08/H1014/39).

### 2.1. Recruitment

This paper focuses on groups identified by AMP as being less likely to seek care and in groups where there was little published literature. We focussed on those with eating disorders, who are homeless, who are asylum seekers, and people from BME communities—Chinese Irish, Somali, and South Asian. Participants were purposively recruited as a convenience sample using links established with local community groups as a part of the AMP programme of work. Such groups offer support to people with distress and life difficulties. Flyers were displayed in the meeting rooms where the groups meet. The participants were invited to participate in this study by the researchers, who described the study and provided an information sheet explaining the study. Key contacts within these organisations also identified potential participants and gave them an information sheet about the study. Participants were people or their carers who identified themselves as having experience of mental health problems or having knowledge of the issues relating to a specific group. Potential participants were informed that they would be reimbursed for their time and travelling expenses (the participants' reimbursement was *£*20 for time and *£*10 expenses). Those who expressed an interest were subsequently contacted by the researcher to organise a suitable time for interview. We aimed to interview at least five people in each group. No diagnostic criteria were used as inclusion or exclusion criteria. A number of interviews were conducted with people who it became apparent had severe and enduring mental health problems (and were psychotic), so this data was not used in the analysis. 

A total of 34 people from the seven groups were recruited, see Table 1.

### 2.2. Data Collection

Data was collected between June and October 2008. 

Semistructured interviews were used to generate data to gain an initial understanding of the parameters of the access issues for each of the groups. Interviews were conducted once formal written consent has been established and in locations convenient to the participant, where necessary interpreters were used and all the interviews were digitally recorded and transcribed verbatim. 

A semistructured approach was taken using story-telling to explore the following topics: experience of seeking help from the GP/health centre/community services for mental health difficulties and needs, aspects of the service found helpful, issues that prevented access, and suggestion for improvements in terms of services and information. The topic guide was developed in reference to AMP's model [10] for researching access to primary mental health care (see topic guide in the Algorithm 1). Interviews were continued until category saturation was achieved across the dataset.

### 2.3. Analysis

The transcripts were analysed using an iterative approach as the data were collected and this analysis and conclusions led to later work in the AMP programme. Data were then reanalysed by the authors once the data collection was complete. Each author was responsible for the analysis of one or two of the seven groups. Transcripts from each group were interrogated using a thematic framework guided by the following questions (generated from the literature about access to care for mental health problems, particularly the issue of candidacy): how do the respondents frame the problems they are experiencing? What help seeking strategies are employed? What do the respondents perceive the role of their general practitioner to be in relation to their problems? What are the implications of the data for primary mental health care policy and practice? Discussion between authors led to the agreement of themes and all transcripts were then reinterrogated by KB to find commonalities and discrepancies in the themes emerging from across the groups. Discussion between authors on the final interpretation led to agreement of the themes. Both manual coding and NVivo 8 software were used to organise the data.

## 3. Findings

The findings are presented under four main themes which directly relate to access to primary care: (1) conceptualising distress, (2) seeking help, (3) barriers to help seeking, and (4) navigating and negotiating services. Data is presented from transcripts and identified by the interview identifier allocated to the participant (see Table 1). Some of the themes presented relate to a specific group whilst others are cross cutting.

### 3.1. Conceptualising Distress

Respondents conceptualised distress in broad terms, linking this to their current or past life difficulties. Thus, those respondents who had eating disorders or were homeless associated their problems with traumas occurring earlier in their lives.

Underlying all this had been for years and years an eating disorder which had its roots in a series of traumas that I suffered from the age of about 14. At one time I took an overdose, I was raped …. (AED3)

I've had a lot of stress in me life, me mum died when I was young, my brother died, he got killed in a car crash when I was young, mum died when I was 8. Then I went off the rail, I went to prison and all that. (HL3)

For asylum seekers distress was related, understandably, to the reasons they were seeking asylum.This was compounded by their current circumstances of being in the UK.

I left Country X, yeah. I was raped, you know, they raped me but I have a child but it's still in Country X, I don't know if she's safe or she's going to school, I don't want to think about that, you know. (AS2)

Other respondents related to the stress of issues such as being carers or the deaths of their elderly parents or spouses.

So I had me Mum dying, me son taking his GCSEs, I had to get all me things in to a friends house, I had to put J [her son] in to me Mum's house, she's dying and I had to stay with me partner, then after that me and me partner split up, so I had all these things going on. (I5)

Older people especially reported that their low mood was linked to poor physical health and exacerbated by feelings of loneliness and isolation.

When I retired,…. And I'm beginning to feel scared and I sat down and said “Is that me finished now? Is that the end?” End of these things. Then I start looking round and I was suffering from, and then the stroke came on. And from the stroke, mental health came on, when I used to go very angry, very snappy, won't talk to people. And I wouldn't do anything for myself. And then eventually, when I realised he's, the doctor told me I've got depression. (SA2)

How distress is conceptualised is likely to influence the decision to seek help. Some respondents explained their distress by attributing external factors as causative mechanisms and suggested that their feelings were understandable, given their circumstances. However, at a certain point all respondents described realising the need for help.

### 3.2. Seeking Help

It was clear from the findings that the respondents looked for a variety of different sources of help and did not necessarily view the GP as an appropriate source or at least not their first or preferred choice.

#### 3.2.1. GP Not Appropriate

The elderly respondents reported being in regular contact with their GPs but reported not mentioning nonphysical issues such as low mood in their consultations.

I only go to see the doctor for my physical health problems, you know I never talk about the mental or emotion side and I assume that he can't do anything and he won't listen anyway. Because of my age the doctor cannot do much more things on me. If my back pain is not that painful I'm maybe happy but if the pain coming you know I'm a little bit unhappy or depressed. (C4)

Such a response suggests that some people look to their doctor for help with physical health problems but do not consider distress as a legitimate reason to attend the GP.

For others, difficulty registering with a GP led respondents to use the Emergency Department (ED) at a time of need.

I don't have a stable doctor, I don't even know how to register to get a doctor, even in I mean, different cases, I'm always running to the Accident & Emergency. (AS4)

Asylum seekers described obtaining a diagnostic label of a mental health problem and thus help during, or possibly as a result of their claim for asylum, completely by-passing the GP.

I went to the Medical Foundation (Medical Foundation for the Care of Victims of Torture) and she finished and then she write a letter, the letter, yeah and then the doctor write to psychiatry, yes. (AS5)

#### 3.2.2. Crisis Precipitates Help-Seeking

For some participants, for example, women with an eating disorder, the decision to seek help tended to arise once their physical health was seriously affected. It was often made by others, and at a time of crisis.

I took her to the Doctors; he told me that often in these cases, “things should be left, and see how they go”. She then shortly after that started secondary school. I was very unhappy about that comment because I knew what was wrong with her by then. I was sure that she had anorexia. I knew that she wasn't eating and I was concerned because she was so small anyway, that a significant loss of weight was going to be quite detrimental to her health. (AED2, mother)

Similarly homeless people were also likely to ask for help when they were experiencing a crisis of some sort, but would go to their local crisis team or ED rather than the GP. They did not see the GP as the person who could provide care.

“I've had mental health problems for the last nine years and throughout the nine years on numerous occasions I've been to see the crisis team at the Royal and other hospitals.” (HL1)

Thus, particularly vulnerable people, such as those who were homeless and asylum seekers, whose lives and living conditions have little stability and thus perceive problems as crises, are not best placed to access primary care systems designed for more well-established populations.

#### 3.2.3. Importance of Community Support

Key to the Irish respondents receiving help, on arrival in England, was their knowledge of the Irish community network.

But basically we've got … me and my partner, that's my partner that came to London first, we'd not planned on coming to Liverpool, and when we got to London met a few Irish…Like it's a new, if the Irish Centre hadn't got involved, it's … they're sort of taking on people with mental health issues and all kind of drug problems in the past and things like that.” (I2)

The Irish centre was reported to sign-post people to and liaise with Social Services and the Drug Dependency Unit (DDU) but there was little mention of referral to/liaison with the GP. 

Older people across the BME groups described the importance of local community services including council run day centres, third sector services, and groups within their own ethnic communities as being arenas where they could gain respite from their feelings of isolation and low mood.

Yes. And the doctor said to me “well, you've got depression” and he give me some tablets. It helps a little bit but it still didn't take depression away very quickly, or took quite a long time. But day centre is the therapy. (SA2)

Those support workers take her to the elderly persons' centre and go to the Chinese supermarket, when she have the day trip to going out, that's the most happy. (C7)

Likewise, women who cared for their sick relatives described gaining support from third sector services.

Since that day, the lady informed me about this project and I'm really happy, I'm glad that I come here. I feel relaxed. Even now, when she phoned me to take a taxi, I don't feel confident taking a taxi so I said no. They gave me a lot of help, they guided me, they've given me guidance as well. (SA4)

Having a good community network helped me, no doctors helped me you know, no services of any kind, the only erm …. I mean I was lucky … that I just, me Mum was part of this (Irish Centre) and they helped her and that's what guides people through, you stick together you know, so they helped me, they guided me, really guided me but the think without these, I probably would have had a severe nervous breakdown …. (I5)

Thus respondents described third sector support as accessible and vital to their recovery. The GP was not seen as accessible to patients whose lives were perhaps more chaotic, but neither was the GP seen necessarily as the person from whom to seek help by other respondents who consulted with physical health problems but were less inclined to present psychosocial distress.

### 3.3. Barriers to Help-Seeking

Respondents described a range of issues that made seeking help for their distress problematic.

#### 3.3.1. Previous Experiences

The participants who had or cared for someone with an eating disorder reported that their GPs' did not pick up on their concerns, and in some cases had been dismissive. Their eligibility as a candidate for help did not seem to be acknowledged.

I went to the doctor and said to him I think I've got a problem, this was my GP, and took my weight and he said I was very underweight, I was only six stone, and he said to me, it's just a phase, you'll grow out of it and he sent me out the door. (AED5)

People in the homeless group articulated concerns that they had been discriminated against because of their homelessness and addictions.

All I got told is “what do you want me to do about it, it's your own fault for going on it [drugs]”. You know, so the health service, I've got no faith in them at all. (HL3)

Reticence to seek help generally, not only relating to consulting the GP, was linked to a concern that they might be stigmatised if they were seen to have mental health problems.

I didn't want to say I was suffering from depression because probably the community would see me as going mad. (S3)

#### 3.3.2. Communication

The ability to speak English is a key factor in whether someone is able to communicate their problems effectively. Even with the help of an interpreter non-English speakers described how they have been misunderstood or have difficulty understanding.

Everybody speaks to me in English and I don't understand all of it, but if they speak Bengali, I can reply to them. (SA3)

Reticence and barriers to considering eligibility to consult a GP are recursive. They are affected by the respondents' previous experiences associated with concerns that their problems would be dismissed, be deemed their own fault, or result in stigma and or discrimination. A more practical issue was the ability for many people from the BME communities and asylum seekers to communicate effectively in English.

### 3.4. Expectations—Navigating and Negotiating Services

Navigation began with registering with a GP. This was described as problematic by some respondents, particularly asylum seekers who reported difficulties registering with a GP while their claims were being assessed. Others do succeed in registering, but described how their attempts to make an appointment with a doctor were confounded by complex systems that did not offer immediate access and incurred long and expensive phone calls.

In the morning, you have to book an appointment, by that same day. So if you have something which is really pressing and you can't book in the afternoon, you don't have money, you have to have a phone in the house, or you have to use your mobile to top up. (AS1)

Respondents described in detail their attempts to make their distress known to the GP within the consultation. Some respondents with eating disorders perceived GPs as the gatekeeper to other services, but attempts to negotiate a referral were seen as being blocked by the GP. However these respondents reported resourcefulness and persistence in their efforts to seek referrals to what were felt to be scant resources, and they approached multiple agencies as well as repeated visits to their GP.

I also went back to the doctor's, GP, and I asked to be put on the list for the dietician. He told me that I'd probably go to the bottom of the list because the priority was diabetics and people who have high blood pressure or obesity are a priority. But I still haven't received any help whatsoever, so I went to a nutritionist and paid for it myself …. I also went and took a course in nutritional therapy …. (AED5)

One respondent described an encounter when her request for a ‘talking treatment' was met by the instruction from the GP to complete a PHQ-9 (PHQ-9 is a patient health questionnaire using a nine itemed depression scale. It is required by Quality Outcomes Framework (QOF) when a diagnosis of depression is made in UK Primary Care [26].) which was perceived by the person as a barrier to further discussion.

Then me Dad died three months later, and I organised all the funerals, all the probate, I had to organise the house, I sorted J (her son) out and then eventually I went to the doctors because I just couldn't cope and I was really, really crying and I said to the doctor, “I need to speak to somebody”, and he said “Well, that'll take 12 or 13 weeks, fill this form in and bring it back next week and we'll see how depressed you are.” (I5)

Respondents described how the GP responded to their distress by the offer of sick note certification or medication.

S3: Well my GP actually was extremely nice but he was only, but I don't know that I can actually get any other help other than the tablets.

Interviewer: Okay, what would you like to have been offered?

S3: Possibly counselling or other ways of, more information about depression itself.

Thus some respondents, for instance, the women with eating disorders, appeared to actively navigate and negotiate to find the help they wish to obtain. Others described more passivity in not being able to access a practice or, even if a consultation is obtained, they find they are not able to ask for specific help. For all respondents, it was the process of negotiation in terms of what they hoped for and what their GP offered that was problematic.

## 4. Discussion

The purpose of this paper was to provide greater insight into the way people from “hard-to-reach” groups seek and access help for their needs which might relate to mental health problems. 

There are highly diverse and complex reasons why people are deemed “hard-to-reach”. These range from specific mental health problems, such as women with eating disorders, social status such as seeking asylum or being homeless, or being a member of different BME groups who have different cultural needs and expectations of primary care health services. Being a member of one group obviously does not exclude people from also coming under another. Thus, the issues emerging from respondents' interviews in this paper are both diverse and cross-cutting. 

Where, when, and if the problems and distress encountered in everyday life become a mental health problem is uncertain and complex. In order to move beyond this complexity and bring further insights into the barriers to access to primary mental health care, Kovandžić et al. [17] describe a five-staged framework. The notion of *silent suffering* conveys the idea that people do not necessarily voice their distress in terms of a mental health issue or in ways that require the assistance of others. All respondents in our study expressed their distress and needs in relation to their past and current social context and experiences. The notion that depression is a social construct and moulded by the medicalisation of chronic distress or unhappiness [30] thus offers a challenge to the position for the need for treatment for what could be viewed as social-existential variations on life. That is, it raises the possibility that a person's mental health needs may or may not be met within the constraints of a general medical setting. 

Help-seeking is a complex iterative process [20] where the actors, health care seekers, and health care providers have to navigate and negotiate not only their own different conceptual understandings of the issues at hand but a range of structural (institutional and statutory) challenges [18, 31]. Thus, help-seeking involves a high degree of *recursivity*. It is dependent on the meaning an individual derives from their past and current lived experience and their specific interactions with relevant others, such as, family and friends, statutory and third sector personnel, and health care workers. For example, asylum seekers, while very likely to have significant mental and social distress, are more likely to obtain a diagnosis and care as they interact with the Home Office or when they get referred to Medical Foundation or other relevant agencies. This confirms a Canadian study which suggested that the nonuse of mental health services by two immigrant groups reflected their understanding and interpretation of their distress and symptoms, their use of different forms of knowledge, both popular and biomedical, and their belief that the origin of their problem resided in social, economic, and political spheres [32].

The women with eating disorders may acknowledge their problem and of the risks involved, but they have more personal agency and therefore control over how and when they decide to ask for specific help. Their awareness for the need for psychosocial intervention is perhaps known to them well in advance but acted upon only at a stage when their condition is recognised by others as needing attention.

In line with previous research [28], the older Chinese participants did not necessarily view their low mood in terms of their eligibility for treatment other than indirectly through relief of their physical symptoms. Deciding to remain silent also appears to be related to fear of discrimination and stigma, especially for people from across the BME groups. Other literature [33, 34], however, suggests whilst people from BME groups provide multifaceted theories of causality and attribution of mental health problems, they are not unfamiliar with the concept of depression. In addition, accounts of help-seeking strategies included prior knowledge of medical and nonmedical “treatments” and talking therapies which may be available to deal with distress. It is likely that sociocultural differences in the way people from BME communities communicate their problems impact on the consultation.

At a more basic level, a person's fluency in English or the ability of a GP to work effectively with an interpreter are also highly important. Past experiences of discrimination have resulted in some of the homeless respondents mistrusting and therefore by-passing their GPs altogether. The possibility that some people feel they do not deserve help as described in other research [29] was not identified in our study.

Common to all seven groups, despite their differences, was what they expected or hoped for from their consultation with the GP. This included their doctor being willing to listen, to refer, or be in liaison with the appropriate specialist services including talking treatments. None of our respondents appeared to go to their GP solely for a medical diagnosis of their problem or a biomedical approach to treatment. 

The management GPs can offer to people with mental health problems is limited by set targets and priorities for specific health problems [35]. The structure of GP consultations assume that diagnostic and management decisions can be made within an average of ten minutes, decisions which are increasingly likely to be constrained or driven by a plethora of clinical guidelines [36]. These restrictions may make it hard for GPs to respond appropriately to highly complex needs. Thus Leydon et al. [37] describe how the requirement to carry out a severity questionnaire such as the PHQ-9 can be seen by GPs as reductionist and unhelpful in some consultations. This situation lends itself to medicalisation [25, 26, 30] and suggests a need to reconfigure primary mental health care within a more overtly social and pluralistic model [17, 38] where GPs are members of a broad range of locally based services. 

This resonates with our respondents' desire for locally based statutory and third sector social services rather than the label or depression and biomedical treatment with antidepressants. Kokanovic et al. [39] talk in terms of a *collision* between the person in distress and current medical practice. That GPs would like to offer an alternative approach which was reported by the Mental Health Foundation [40] suggesting that, while they acknowledge the role of talking treatments including mindfulness, some GPs fall back to a more straight forward approach that lends itself to medicalisation [30] due to lack of alternative approaches in practice. 

Whilst many of these lessons could be applicable for anyone attempting to consult primary care with a possible mental health problem, they are perhaps of more consequence for people whose lives are chaotic (homeless, asylum seekers), who are isolated (elderly, BME), or whose approach to illness is such that help is sought at a time of crisis (AED, homeless). So whilst many people consulting GPs with depression can be reasonably managed according to NICE guidelines [41], with referral into IAPT services [42] this may not be appropriate or acceptable for people in marginalised groups. The IAPT initiative [42] particularly needs to be able to offer interventions which are flexible and responsive to the needs of different groups of patients.

## 5. Strengths and Limitations

The data from which this paper is derived were gathered as part of a qualitative study within the AMP programme to gain understanding of the key issues relating to access to primary health care for hard-to-reach groups. The main strengths of this paper are its ability to provide first-hand data from members of marginalised communities, approached purposively through local third sector organisations, and the increased trustworthiness of the analysis through participation of researchers from a number of disciplines [43]. Some interviews were conducted using an interpreter; while there may be concerns over the accuracy of translation, this also strengthens the study, by adding voices that would otherwise be missed.

Data analysis was conducted in two stages: the initial analysis was conducted parallel with data collection by the research team members conducting the interviews. Reanalysis of the dataset reported in this paper was conducted by original researchers and new researchers, against a framework developed from the literature and previous work within AMP [10]. Whether this could be defined as secondary analysis [44] is open to argument. The data had been collected to explore issues around access to care for mental health problems and some of the researchers involved in collecting the data were involved in the reanalysis, so it could be argued that the analysis here was a progression of the primary analysis. However, the data were interrogated using a new conceptual framework [17] and researchers new to the data were involved, which arguably makes the process a secondary analysis. The involvement of new researchers may have improved the quality of the analysis or, by removing analysis from the initial data collection, may have reduced the validity of the analysis. 

An advantage of secondary analysis is that it reduces respondent burden and research resources (reducing the repetition of repeated data collection), while multiplying original respondents' contributions and primary researchers' efforts. Van der Berg (2005) suggests that secondary analysis permits only a limited range of possibilities for research but acknowledges that for some research goals, secondary analysis may constitute a very fruitful alternative provided that sufficient contextual information is made available to the researchers [45]. We would argue that the analysis presented here has made an effective and efficient use of precollected data.

Our recruitment strategy, sampling from existing community groups, and relying on flyers to publicise the study and the group leaders to invite potential participants, meant that we do not have any information on how many people were approached in order to achieve our target. We did not achieve a full range of sociodemographic variables within each of the groups involved: for instance, all the Chinese participants were over 60. There is a gender bias in that the majority of our participants were female. We experienced difficulty recruiting participants from the Somali community group, despite intensive efforts. 

We focussed this work on hard-to-reach groups identified as important within the AMP study, and we recognise that there are other marginalised groups which we did not investigate. It is possible that participants selected via the community groups may have, out of loyalty, emphasised the support they had received from these agencies over and above other services. A nominal fee of *£*20 and travel expenses were paid to each participant, and it is not known whether this might have affected the recruitment and participants' motives for taking part.

## 6. Implications

This study illuminates the complex reasons why people from “hard-to-reach” groups may not conceptualise their distress as a biomedical problem to present to the GP, and hence may not present or present to alternative services. In addition, the barriers to accessing primary care by people from marginalised groups when distress is recognised by the person, and help-seeking attempted, are described. There is an extensive literature on the need to GPs to improve their skills in detecting distress and mental health problems [22, 23], but it is clear that people may not even get as far as a GP consultation to present their distress because of perceptions of what primary care can offer, which may be based on previous experiences. 

So what are the implications of this study for policy and practice? We suggest that interventions are needed at a number of levels.

It is vital to enable people with distress to present their problems to primary care, so a public health campaign alerting people to the appropriateness of such a presentation, allowing people to feel candidates for care, is required. Such public health messages need to take account of cultural issues and health literacy in hard-to-reach groups. 

At a practice level, awareness raising and training of primary care practitioners in cultural competence is vital [46], emphasising how it specifically relates to the lives and experiences of all groups within their practice population. Practitioners need to attempt to enter the “life-world” of their patients and understand the patient's language representing distress, as well as how the broader social context can impact on people. It is important that primary care practitioners recognise the impact of economic and social deprivation, isolation, and loneliness on psychological health. Practitioners need to understand the broader causes and context(s) of depression and offer alternative treatment options to the biomedical model. In particular, recognition of the roles played by social isolation in suffering and the need to “be with people” suggests that interventions should address such issues. 

GPs need knowledge of third sector services, community groups, and referral pathways, in order to support people in accessing more acceptable support to help address their problems. This will require to improve links between health, social, and third sector care. There may be opportunities for innovative commissioning decisions by GP-led commissioning consortia [47] working with local populations, to enable more psychosocial interventions to be available for patients with distress which may be conceptualised as common mental health problems. Whilst primary care is driven by biomedical protocols [26], however, opportunities for GPs to avoid medicalising people with distress may be limited.

## Figures and Tables

**Algorithm 1:**
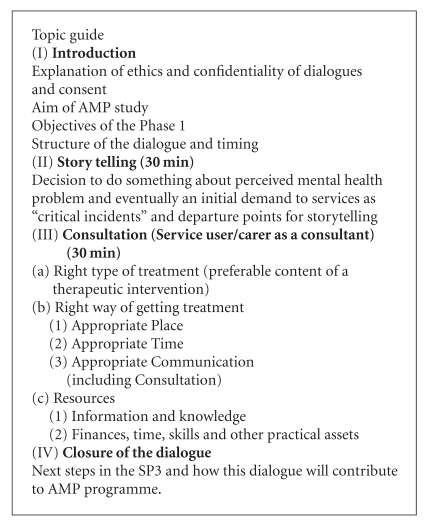
Topic guide.

**Table 1:** Respondent details.

Group		Gender	Age	Education	Employment
Eating disorders					

AED1		F	29	College	Professiona dancer
AED2	Carer	F	56	GCEs	Foster carer
AED3		F	44	Graduate	Teacher
AED4	Carer/AED recovered	F	18	A levels	Police officer
AED5		F	24	Graduate	Unemployed

Homeless					

HL1		M	Missing data	Missing data	Unemployed
HL2		M	Missing data	Missing data	Unemployed
HL3		M	34	GCSEs	Unemployed
HL4		F	43	GCSEs	Unemployed
HL5		M	40	Left school at 13 years	Unemployed
HL6		F	Missing data	Missing data	Unemployed

Asylum Seekers					

AS1		F	39	Graduate	Missing data
AS2		F	33	College	Missing data
AS3		F	43	Masters	Volunteer
AS4		M	26	A levels	Volunteer
AS5		M	18	GCSEs	Studying

Chinese					

C1	Interpreted	F	60	Attended school between ages of 11 and 16	Missing data
C4	Interpreted	F	81	No schooling	Agriculture and catering (retired)
C5	Interpreted	F	77	Disrupted schooling between ages of 12 and 21	Cook/waitress (retired)
C6	Interpreted	M	76	Left school at 12 years	Cook (retired)
C7	Interpreted	F	67	Left school at 14 years	Home-maker

Irish					

I2		M	31	GCSE	Unemployed
I3		F	32	Left school at 11 years	Unemployed
I4		F	59	PostGraduate diploma	Freelance consultant
I5		F	46	Missing data	Support Worker

South Asian					

SA1	Interpreted	F	63	Missing data	Machinist/Market trader
SA2		M	72	Missing data	Clothes designer (retired)
SA3	Interpreted	F	56	Left school at 12 years	Home-maker
SA4	Interpreted	F	49	Left school at 10 years	Unemployed
SA5		F	28	GNVQ	Home-maker

Somali					

S1	Interpreted	F	58	No schooling	Not working due to health problems
S2		F	34	Left school at 12 years	Missing data
S3		M	40	College	Unemployed

## Appendix

See Algorithm 1.
